# Stress responses, social support and death care experience among spouse caregivers of young and middle-aged patients with terminal cancer: a mixed-methods analysis

**DOI:** 10.3389/fonc.2024.1464132

**Published:** 2024-11-14

**Authors:** Shaoyuan Xu, Guizhen Weng, Xiaoyan Chen, Lina Liu, Huan Chen

**Affiliations:** ^1^ Department of Oncology Nursing, Fujian Maternity and Child Health Hospital College of Clinical Medicine for Obstetrics & Gynecology and Pediatrics, Fujian Medical University, Fuzhou, China; ^2^ Department of Oncology Nursing, Fujian Medical University Union Hospital, Fuzhou, Fujian, China; ^3^ School of Nursing, Fujian Medical University, Fuzhou, Fujian, China

**Keywords:** cancer, spouse, stress response, social support, death care experience

## Abstract

**Background:**

Faced with cancer patients in the near-death stage, spousal caregivers may experience a series of stress reactions and have a high risk of suffering from physical and psychological problems. Good social support can help alleviate stressful reactions.

**Objectives:**

To investigate stress responses and social support among spouse caregivers of young and middle-aged patients with terminal cancer in the near-death stage, and to explore the death care experience of spouse caregivers.

**Methods:**

A prospective mixed-methods study was conducted. Questionnaires of stress response questionnaire and social support rating scale were used to investigate stress responses and social support of spouse caregivers. And semi-structured interviews were conducted to explore the death care experience of spouse caregivers in at a university-affiliated hospital in China.

**Results:**

Spouse caregivers with higher social support scores were significantly more likely to have poor stress response. Meanwhile, whether the spouses had alternative care for other dependents, the number of venous pathways and instruments in/on the patient’s body significantly affected the spousal stress response. Among them, spousal social support was the best influencing factor to predict spousal stress response. Four qualitative themes of the death care experience were identified. Theme 1: Psychological feelings of spouses caregivers when they care for the patients’ physical function. Theme 2: Psychological feelings of spouse caregivers when they communicated with the patients. Theme 3: Psychological feelings of spouses caregivers when they will being widowed soon. Theme 4: The focus of life shift, and life concept change.

**Conclusion:**

Overall, spousal stress response was statistically affected by alternative caregivers for spouses, spousal social support, and the number of venous pathways and instruments in/on the patient’s body. Among that, social support was the best influencing factor to predict the stress response. Meanwhile, spousal caregivers was distressed and felt deeply fear, wronged and helpless deep and when facing the patients’ dying symptoms and communicating with patient, and reflected on the essence of life, and changing the concept of life.

**Implications for practice:**

Medical staff should pay special attention to spousal caregivers’ physical discomfort and improve spousal social support, and provide targeted information and assistance to decrease spousal stress response in the near-death stage of cancer patients.

## 1 Introduction

Malignant tumours are among the most serious public health problems that threaten human health in the 21st century. According to a report by the China National Cancer Center, in 2022, there were approximately 4.82 million new cancer cases in China and the number of deaths will be approximately 3.21 million (1). Cancer is the leading cause of death among the urban residents in China. In recent years, the incidence of cancer in different age groups has shown an upward trend, and the group of patients with cancer has gradually become more frequent in younger patients (2, 3). In the United States, approximately 90,000 adolescents and young adults are diagnosed with cancer (3). Compared with older adults, young and middle-aged patients with cancer often do not have typical clinical symptoms in the early stages of cancer (4). However, once discovered, they often progress to late stages of cancer, which is characterised by rapid progression, early metastasis, high malignancy, and poor prognosis (4, 5). Meanwhile, symptoms indicating death, such as coma, severe vomiting, dyspnoea, and bleeding, may cause severe distress upon patients, and simultaneously elicit a series of stress responses in spousal caregivers of patients with terminal cancer (4). This was a major negative life event for both patients and their spouses (6–9). If patients have terminal cancer, most may lose their self-care ability and depend completely on others for daily care. Young and middle-aged populations may experience more problems after illness comparing to older adults, such as depression, severe psychological distress, and suicidal thoughts (10). Young and middle-aged patients with cancer and their family members need to cope with not only physiological and psychological pain, but also with changes in social roles and lifestyles when facing the disease and treatment process (5). Marriage relationship was the main source of support for young and middle-aged patients with cancer who were married (11). Spouses often played an active role in medical decision-making and provided emotional support for patients, and also had a higher nursing care burden than other types of caregivers (11). Studies found that compared with healthy people, the spouses caregivers of cancer patients were more likely to have anxiety and depression symptoms (8, 11, 12), which indicated that the psychological problems of spouses caregivers needed urgent attention. Moreover, there was a significant association between the psychological health status of cancer patients and the psychological health of their spouses (11, 12). Therefore, attention should be paid to psychological, social, and psychological pain in young patients with cancer and their spouses (13) to provide care and companionship to their loved ones during the end-of-life process (14).

Stress responses refer to physiological, psychological, and behavioural reactions to stressful events (15). The spouse is the closest companion during surgery, chemotherapy, disease improvement, recurrence, and changes when the patient is diagnosed with cancer (4, 5). Every fluctuation during treatment was a positive or negative event for the spouse. Faced with cancer patients in the terminal stage, spousal caregivers may experience a series of stress reactions and have a high risk of suffering from physical and psychological problems (8, 9). Spousal caregivers often played an active role in medical decision-making and provided emotional support for cancer patients and nursing care support (11). Affected by the uncertainty of the patient’s condition and prognosis, the work and social activities of spousal caregivers changed, as well as the economic burden and psychological pressure of them were also heavy. Good social support could help alleviate stressful reactions. However, few studies have focused on stress responses and social support among spousal caregivers of young and middle-aged patients with terminal cancer (16). Therefore, understanding stress responses and social support will help healthcare workers provide more comprehensive support and care for patients with terminal cancer and their spousal caregivers or other families. This study aimed to use a quantitative study design to investigate stress responses of spousal caregivers who were spouses of young and middle-aged patients with terminal cancer and their influencing factors, including social support, based on the above mentioned research results.

Compared with quantitative study method, qualitative study method focuses on exploring and interpreting the deeper content of the research objects. Due to the fact that the care experience had strong subjectivity, individuality and complexity, which could not be used scales to complete express and embody care experience, and spousal caregivers was in a special period of the loss of their lover, a semi-structured interview could more in-depth and comprehensive understand the individual in a specific cultural background detailed, dynamic experience and feelings. So a qualitative study of Phenomenology was also used in this study to further explore the death care experience of spousal caregivers. This mixed-methods study aimed to more comprehensive grasp of the physical, psychological, social and economic feelings and reactions of spousal caregivers. And then this study attempted to provide a basis and guidance for clinical medical staff in formulating humanistic nursing intervention measures for this population.

## 2 Methods

### 2.1 Study setting and design

This mixed-methods study used survey data and semi-structured interviews. It was based on the graduate student project’s qualitative and quantitative data, including more targeted questions about stress responses and death care experiences among spousal caregivers of young and middle-aged patients with terminal cancer. This study was approved by the Human Research Ethics Committee of the Research Hospital (No. 2023YK070). A total of 201 spousal caregivers were recruited by a convenience sampling method from the Department of Oncology of a university-affiliated hospital in China between January 2022 and December 2023. Among them, 10 spousal caregivers withdrew from the study due to the intense mood or fear of taking up the care time for their spouses, and 5 spousal caregivers were removed from this study due to the fact that their spouses’ condition of terminal cancer had improved and left the dying period. Finally, totally of 186 spousal caregivers completely participated in the study.

### 2.2 Participants

The eligibility criterion for enrolment in this study included (1) age between 18 and 59 years, (2) patients who met the pathological diagnosis of a malignant tumour, and (3) patients in the near-death stage (the medical staff jointly determined that the patient was near death). The exclusion criterion were as follows: (1) divorced, widowed, and not remarried, and (2) the survival period of patients could not be evaluated or determined.

Meanwhile, the inclusion criterion of spouse caregivers also included: (1) taking primary care and medical decision-making responsibility during the patient’s diagnosis, treatment, and care for at least one month; (2) when the patient was in the near-death stage, the spousal cumulative daily care time was more than eight hours; (3) without cognitive disorder which was identified clear logic thinking and barrier-free communication with researchers; and (4) ability to complete the questionnaire independently or under the guidance of the researcher. The inclusion criterion of spouse caregiver for semi-structured interview were in the same with above. We purposefully sampled participants for interviews from recruit spouse caregiver.

### 2.3 Sample size

The sample size for the quantitative data was calculated based on a calculation of five times to ten times the number of items in the stress response questionnaire (28 items) (17), so the sample size of this study was about 140 to 280 cases. Meanwhile, the sample size was at least above 300 in the field of social and behavioral science, however, most of the studies did not reach it, so the sample size of around 150 was enough (18). Considering the unpredictable number of deaths among young and middle-aged patients with terminal cancer during the study period and the limited source of patients, so the study sample size was planned to be 150 cases at least.

For the qualitative study, sampling was terminated when repetitive data from the participants and the absence of new topics during data analyses (data saturation) were observed after three consecutive interviews, sample size was about 10-20 (19). In this study, qualitative sample size was 10 cases achieving data saturation, including 5 male and female spouse caregivers, aged between 29 and 59.

### 2.4 Procedure

All participants completed the questionnaire after providing consent, and a subset completed a semi-structured interview a few days after completing the questionnaire. Prior to the semi-structured interview, each participant verbally repeated the voluntary and informed consent to participate. The interviews explored the participants’ questionnaire responses in greater detail, with a rationale and further details about their questionnaire responses.

### 2.5 Data collection

A questionnaire survey conducted following the STROBE statement was used to collect data. The investigators were trained nurses. Informed consent was obtained from all participants prior to the study. The investigator completed a general information survey of the patients and spouses, and the spouses completed the stress response and social support questionnaires. After completing the questionnaires, on-site verification was conducted.

Besides, this qualitative study was also conducted following conducted following the COREQ statement. Before the interview, the researcher made an appointment with the interviewees, and informed them of the general content and direction of the upcoming interview to prepare them. Due to the special limitation that the cancer patients were in the near-death stage, researcher should communicate well with spousal caregivers according to the condition of each patient, and tried to conduct the interview when the family member replaced to spousal caregivers to care patients, otherwise researcher should ask other nurses in the ward to help with the care. Spousal caregiver were one-by-one invited by the nursing head of the Oncology Department (Weng) to participate in audio-recorded interviews. The interviews were conducted in the office of the Department of Oncology where keep the indoor light soft, prepare drinking water, paper towels and other items. It lasted between 30 and 60 minutes. The researcher (Xu) started the interview according to the current condition and symptoms of their spouse as the entry point, and gradually entered into the dying care experience according to the proposed interview guide, so that the interviewees could explain their feelings, ideas and experiences in a more comfortable, relaxed and trusting situation. In the process of interview, researcher (Xu) tried to avoid the use of professional terms, and payed attention to linguistic and non-verbal communication skills, and responded to the elaboration content of interviewees appropriately to promote their further narration. However, in the process of communication with interviewees, no hint, induction and critical reply should be made, so as to avoid deviation. Within 24 hours of the interview, the researcher converted the recordings into text according to the original narrative of the interviewees and input them into NVIVO software. To maintain the originality of the data, they did not delete or modify it at will.

### 2.6 Measures

#### 2.6.1 Quantitative

##### 2.6.1.1 General questionnaire

A general questionnaire was designed by our study team based on literature review, oncologist guidance, and clinical experience. The content included (1) general information about spouses (gender, age, marital age, and economy), (2) spousal care information (care duration, sleep time, and whether there are family substitutes), and (3) general information about the patient (age, disease, cancer age, and disease diagnosis).

##### 2.6.1.2 Stress response

The stress response questionnaire was designed by the Department of Medical Psychology, Zhejiang University, China (15) to assess the degree of individual physical and mental responses to stressors, including physiological, psychological, and behavioural response dimensions, with 28 items. Each item is scored on a 5-point Likert scale. The total score on this questionnaire ranges between 28 and 140 points. The better the total score, the more intense the individual stress response. The Cronbach’s α coefficient was 0.902, and the test-retest reliability was 0.913 (15). Cronbach’s α coefficient in this study was 0.897.

##### 2.6.1.3 Social support rating scale

The Social Support Rating Scale compiled by Xiao Shuiyuan is widely used in the medical field in China (20). This questionnaire was mainly used to evaluate individual objective support, subjective support, and the degree of utilisation of social support dimensions with 10 items. Each item was scored on a 4-point Likert scale. The total score on this questionnaire ranged from 10 to 40 points. A total score of < 33 indicates low social support, 33−45 general social support, and > 45 indicates high social support. A higher total score indicates a higher level of social support. The Cronbach’s α coefficient was 0.890−0.940, and the test-retest consistency was 0.92 (20). Cronbach’s α coefficient in this study was 0.911.

#### 2.6.2 Qualitative

Before compiling the interview guide, the relevant literature was firstly inquired (22–24), and the preliminary interview guide was prepared based on relevant literature and the results of the quantitative study. And then, two oncologists and two researchers in the field of hospice care were invited to adjust and revise the interview guide. After that, researcher (Xu) conduct a pre-interview of one spouse caregiver and revised the interview guide again according to the problems existing in the pre-interview. And the pre-interview results were not included in this study. Data analysis after each interview informed adjustments to the guide, ensuring its continuous refinement until reaching a stable version. The main questions of the interview guide were as follows.

- Could you talk about your spouse’s previous role in your family?
- Could you describe your affection for your lover?
- How do you feel about your spouse’s physical and psychological changes during the dying period?
- Could you describe the main actions you took to care of your spouse during this period? How do you feel internally when doing these things?
- What burdens, difficulties, and obstacles do you experience during the care process?
- What did you think of your spouse during the dying period?

A subset of ten spouse caregivers completed a semi-structured interview on their experiences of death care for young and middle-aged patients with terminal cancer. Semi-structured interviews, lasting an average of 30 minutes, were conducted by experienced qualitative researcher (Xu), audio-recorded, professionally transcribed verbatim, and verified for accuracy. The participants were asked about their reflections after completing the questionnaire.

The quality control of this qualitative study included: (1)All data collection at this phase was conducted by the researcher (Xu); (2) During the interview, the researcher (Xu) shall not have any suggestion or guidance, and try to let the interviewees speak freely as relaxed as possible. (3) Researcher carefully checked unclear questions in the data and asked the interviewees for feedback to increase the accuracy and completeness of the data; (4) In the process of research, researchers should repeatedly think about themselves, discuss each other or actively consult oncologists and researchers in the field of hospice care, and timely adjust the details of the research implementation process according to the actual situation. (5) In the text transcription process, researchers (Xu and Weng) independently transcribed text and checked each other with the transcription text to ensure accuracy and completeness. Meanwhile, they should record in strict accordance with the original description of the interviewees.

### 2.7 Data analysis

#### 2.7.1 Quantitative

SPSS software (version 25.0) was used for the statistical analysis. General information is presented as the mean ± standard deviation for continuous variables and frequencies (%) for categorical variables. A t-test or one-way analysis of variance was used to evaluate and compare the different general information and stress responses. Spearman’s analysis was used to estimate the correlation between social support and stress responses. Furthermore, multiple linear regression analysis was conducted to identify the factors influencing spousal stress response by incorporating all significant variables from the aforementioned analysis. The independent variables was the variables with statistical significance in the univariate analysis and spousal social support. Regression coefficients (β) and 95% confidence intervals (CIs) were estimated as the independent variables in the regression analysis. Statistical significance was set at *P* < 0.05.

#### 2.7.2 Qualitative

Interviews were imported into NVIVO software (version 9.0) for analysis. Two researchers (Weng and Xu) independently analysed all qualitative data using the Colaizzi phenomenological analysis method for content analysis (25). This method includes seven iterative steps: (1) reading all the original description records carefully; (2) analysing and extracting important statements; (3) coding the recurring and representative points; (4) collecting and summarising the coded points; (5) summarising detailed and accurate points; (6) identifying similar points, refining, and gradually sublimating into the theme; and (7) returning to the respondents for verification. All transcription text data were read, re-read, and coded separately by two researchers (Weng and Xu) following Phenomenology approach and simultaneously with data transcription. Any discrepancies in themes naming or data placement were resolved and discussed by mutual agreement between the two researchers (Weng and Xu). Similar themes naming and controversial themes were resolved through discussions by the research team. And then, the transcripts were then translated into English by a professional translator.

## 3 Results

### 3.1 Participants

In this study, the average age of 186 spouse caregivers of young and middle-aged patients with terminal cancer was 47.80 ± 8.15; more than half of them were female (58.1%). The demographic characteristics of the spousal caregivers are presented in **Table 1**. In addition, the average age of 186 young and middle-aged patients with terminal cancer in this study was 48.67 ± 8.49; more than half of them were male (58.1%). The demographic characteristics of the patients are shown in **Table 2**.

**Table 1:** Demographic characteristics in spouses of young and middle-aged patients with terminal cancer, and the univariate analysis for stress response of spouses (n=186).

Characteristic	Number (%)	$\bar{\text{x}}$ ± s	*F*/*t*/r value	P value
Gender				
male	78 (41.9)	61.46 ± 21.88	-2.981	0.003
female	108 (58.1)	70.86 ± 20.74		
Age (years)				
< 45	66 (35.5)	72.12 ± 22.41	2.461	0.015
45 to 59	120 (64.5)	64.06 ± 20.79		
Marriage age (years)				
< 3	8 (4.3)	83.13 ± 22.71	5.927	0.001
3-5	19 (10.2)	78.05 ± 21.31		
6-10	30 (16.2)	73.07 ± 20.43		
> 10	129 (69.4)	62.84 ± 20.78		
Highest Level of Education				
Elementary school and below	35 (18.8)	74.97 ± 22.18	1.963	0.102
Junior High	72 (38.7)	65.19 ± 21.69		
High school and secondary school	31 (16.7)	67.35 ± 23.38		
Associate and undergraduate	37 (19.9)	65.14 ± 19.77		
Master’s degree and above	11 (5.9)	57.36 ± 16.30		
Religious beliefs				
Yes	55 (29.6)	66.25 ± 22.39	-0.270	0.787
No	131 (70.4)	67.20 ± 21.44		
Occupations				
Unemployed	74 (39.8)	68.49 ± 22.29	0.324	0.723
Employed	100 (53.8)	65.81 ± 21.54		
Retirement	12 (6.5)	66.50 ± 19.85		
Family income per year				
< 100,000 Yuan (13,816.5 Dollar)	40 (21.5)	80.90 ± 24.67	8.157	0.000
100,000 Yuan to 200,000 Yuan (13,816.5 Dollar to 27,633 Dollar)	46 (24.7)	65.13 ± 19.59		
> 200,000 Yuan and ≤ 250,000 Yuan (> 27,633 Dollar and ≤ 34,541.5 Dollar)	54 (29.0)	62.83 ± 19.18		
> 250,000 Yuan (34,541.5 Dollar)	46 (24.7)	61.35 ± 18.89		
Current place of residence				
Rural Area	69 (37.1)	75.07 ± 23.64	10.534	0.000
Town	34 (18.3)	61.94 ± 18.04		
Downtown	83 (44.6)	62.18 ± 19.39		
Duration of continuous patient care				
< 3 months	23 (12.4)	63.83 ± 24.70	2.456	0.065
3-12 months	85 (45.7)	64.66 ± 21.67		
13-36 months	51 (27.4)	66.76 ± 19.78		
> 36 months	27 (14.5)	76.96 ± 20.67		
Average sleep duration per day over the past week (hours/day)				
4 or less	23 (12.4)	71.35 ± 25.18	1.115	0.330
5-6	104 (55.9)	67.65 ± 20.23		
7 or higher	59 (31.7)	63.90 ± 22.64		
Average number of hours per day spent caring for patients in the past week (hours/day)				
9 or less	47 (25.3)	65.47 ± 22.73	0.178	0.837
10-12	86 (46.2)	67.01 ± 20.81		
13 or higher	53 (28.5)	68.06 ± 22.42		
Have any other family members taken care of the patient in the past week.				
0	11 (5.9)	79.36 ± 18.70	11.158	0.000
1 to 2 family members	127 (68.4)	70.20 ± 21.93		
3 or higher family members	48 (25.8)	55.40 ± 16.84		
Social support score	186(100)	40.86 ± 8.21	-0.503	0.000
Subjective support	186(100)	20.69 ± 5.52	-0.365	0.000
Objective Support	186(100)	11.69 ± 3.30	-0.447	0.000
Support utilisation	186(100)	8.48 ± 2.22	-0.285	0.000

**Table 2:** The univariate analysis of young and middle-aged patients with terminal cancer ‘ demographic characteristics for spouses’ stress response (n=186).

Characteristic	Number (%)	$\bar{\text{x}}$ ± s	F/t value	P value
Gender				
male	108 (58.10)	70.86 ± 20.74	2.981	0.003
female	78 (41.90)	61.46 ± 21.875		
Age (years)				
< 45	59 (31.7)	70.08 ± 21.30	1.361	0.175
45 to 59	127 (68.3)	65.45 ± 21.77		
Highest Level of Education				
Elementary school and below	34 (18.3)	70.59 ± 22.54	0.928	0.449
Junior high school	77 (41.4)	66.52 ± 20.64		
High school and secondary school	45 (24.2)	68.58 ± 24.62		
Associate and undergraduate	23 (12.4)	62.74 ± 17.87		
Master’s degree and above	7 (3.8)	56.57 ± 19.48		
Medicare payment method				
Self-pay	9 (4.8)	70.78 ± 26.68	1.969	0.143
New rural cooperative medical care system/urban medical insurance system	89 (47.8)	69.83 ± 20.66		
Provincial and municipal medical insurance	88 (47.3)	63.65 ± 21.90		
Disease diagnosis				
Lung Cancer	47 (25.3)	66.51 ± 22.55	1.188	0.309
Stomach Cancer	32 (17.2)	62.69 ± 22.80		
Bowel cancer	36 (19.4)	68.14 ± 22.76		
Breast cancer	17 (9.1)	66.35 ± 19.98		
Hepatopancreatic biliary malignancy	14 (7.5)	62.14 ± 19.75		
Tumours of the reproductive system	13 (7.0)	65.85 ± 20.03		
Lymphoma	10 (5.4)	65.30 ± 17.88		
Carcinoma of oesophagus	8 (4.3)	86.25 ± 20.01		
Others	9 (4.8)	73.89 ± 18.87		
Duration of Cancer				
< 3 months	19 (10.2)	73.05 ± 20.69	2.763	0.043
3-12 months	84 (45.2)	69.80 ± 23.13		
13-36 months	55 (29.6)	64.85 ± 20.96		
> 36 months	28 (15.1)	58.18 ± 16.40		
How many venous pathways were in the patient body				
1	81 (43.5)	60.69 ± 21.33	8.666	0.000
2	58 (31.2)	67.84 ± 22.07		
3 or higher	47 (25.3)	76.51 ± 18.17		
Number of instruments were used on the patient body				
1	77 (41.4)	60.25 ± 19.56	12.056	0.000
2	55 (29.6)	65.44 ± 23.42		
3 or higher	54 (29.0)	77.94 ± 18.45		

### 3.2 Quantitative results

#### 3.2.1 Univariate analysis of spouses’ and patients’ demographic characteristics for spousal stress response

The results of the univariate analysis showed that spousal stress responses were significantly different between spouses’ gender, age, marital age, family income per year, current place of residence, and having any other family member caring for the patient in the past week (*P* < 0.05, **Table 1**). Furthermore, there was a negative and moderate correlation between spousal stress response and spousal social support (r = -0.503, *P* < 0.05, **Table 1**). Additionally, spousal stress responses were significantly different in terms of patients’ gender, cancer duration, number of venous pathways in the patient’s body, and number of instruments used (*P* < 0.05; **Table 2**).

#### 3.2.2 Spousal stress response questionnaire scores

In this study, the spousal stress response questionnaire score was 66.92 ± 21.66 (**Table 3**). The scores of psychological reaction dimensions was highest, followed by he scores of physiological reaction dimensions (**Table 3**). However, the scores of behavioral response dimensions was lowest (**Table 3**).

**Table 3:** Spousal stress response questionnaire scores.

Variables	Spouses (n=186)
Physiological reactions	19.89 ± 6.00
Psychological reaction	30.47 ± 12.00
Behavioral response	12.59 ± 5.10
Total stress response Score	66.92 ± 21.66

#### 3.2.3 Multiple linear regression analysis of influencing factors of spousal stress response

Multiple linear regression analysis was conducted to further explore the relationship between spousal social support and spousal stress response. In the unadjusted model (R^2^ = 0.212, F = 49.393, *P* < 0.01), spouse caregivers with higher social support scores were significantly more likely to have poor spousal stress response (*β* = -0.460, 95% CI: -1.461 to -0.820, **Table 4**). After adjusting for spouses’ gender, age, marital age, family income per year, current place of residence, having any other family member caring for the patient in the past week, and in terms of patients’ gender, cancer duration, number of venous pathways in the patient’s body, and number of instruments used (adjusted R^2^ = 0.358, F = 11.296, *P* < 0.01), spouse caregivers with higher social support scores remained more likely to have poor spousal stress response (*β* = -0.292, 95% CI: -1.041 to -0.408, **Table 4**). Meanwhile, the results also showed that three influencing factors statistically affected spousal stress response: whether the spouse had alternative care for other dependents (*β* = -0.217, 95% CI: -7.957 to -2.046), the number of venous pathways in the patient’s body (*β* = 0.149, 95% CI: 0.318 to 7.640), and the number of instruments on the patient’s body (*β* = 0.15, 95% CI: 0.290 to 7.536) (**Table 4**). Overall, among these influencing factors, social support was the best influencing factor to predict the stress response, followed by whether the spouse had alternative care for other dependents (**Table 4**).

**Table 4:** Multiple linear regression analysis of spousal stress response influencing factors (n=186).

Variables		B	SE	Beta	*t*	*P*	95% CI of B		VIF	R^2^ /Adjusted R^2^	F/*P*
							Lower limits	Upper limits			
Unadjusted											
(Constant)		113.249	6.743		16.795	0.000	99.946	126.552		0.212	49.383/0.000
Social Support score		-1.140	0.162	-0.460	-7.027	0.000	-1.461	-0.820	1.00		
Multivariable adjusted											
(Constant)		99.74	10.013		9.961	0.000	79.978	119.502		0.358	11.296/0.000
Spouse	Alternative care for other dependents	-5.001	1.497	-0.217	-3.34	0.001	-7.957	-2.046	1.22		
	Social Support score	-0.724	0.16	-0.292	-4.515	0.000	-1.041	-0.408	1.206		
Patient	Number of infusion lines	3.979	1.855	0.149	2.145	0.033	0.318	7.64	1.389		
	Number of instruments	3.913	1.836	0.15	2.131	0.034	0.29	7.536	1.432		

B, partial regression coefficient; Beta, standardized multiple linear partial regression coefficient; SE, standard error.

### 3.3 Qualitative results

Four overarching themes were identified in the qualitative analysis, as discussed below.

#### 3.3.1 Theme 1: Psychological feelings of spouses caregivers when they care for the patients’ physical function

##### 3.3.1.1 Feeling deeply distressed when spouse caregivers faced the patients’ dying symptoms

Death is often accompanied by physical pain (26). Patients with terminal cancer often experience severe unrelieved pain, gatism, dyspnoea, stress, nonsense, and other terminal symptoms. Their mental and physical states are severely compromised (26). Most young and middle-aged patients with terminal cancer were originally the backbone of the family, and now, the near-death stage of patients has formed a huge contrast with their prior state. As in the most intimate relationship, the accompanying spouse felt uncomfortable and distressed.

‘He used to be in excellent good health before, never even been to the hospital. This disease has changed in many ways. Only a year ago, he was a man in good health, but that day, he vomited a lot of blood in front of me; his chin and clothes were full of blood (choked with sobs). Subsequently, he was lying in bed and became very weak. We looked uncomfortable and very awful (crying)’. (Spouse 9)

‘He is what he is now. He is so thin that it seems that there was only a layer of leather bag left in his body. What we see is very upsetting and heartrending’. (Spouse 10)

##### 3.3.1.2 Feeling deep fear when spouse caregivers touched the machine and pipeline on the body of patients with terminal cancer

To maintain the life and treatment of patients, healthcare workers must use various instruments and drainage pipes for monitoring. This is a powerful internal stimulus for spouses and their family members. When they take care of various tasks such as wiping, dealing with urine, turning over, and other matters, they often worry that their carelessness or ignorance will cause problems in the machines and pipelines, which will endanger the lives of patients.

‘My husband now has an abdominal tube on the left side of his abdomen, a thoracic tube in the right of the chest, and a PICC tube on the hand for venous transfusion. Sometimes, a lien needle tube is placed on the hand during blood transfusion. It is scary that he is filled with tubes in the body. When we wipe him, we are afraid of touching it off or distorting it off’. (Spouse 1)

#### 3.3.2 Theme 2: Psychological feelings of spouse caregivers when they communicated with the patients

##### 3.3.2.1 Feeling deeply wronged and helpless when spousal caregivers communicated with patients

Patients with terminal cancer can experience a full outbreak of terminal symptoms with obvious malaise (27). On one hand, they are forced to be confined to the bed and cancel their interaction and contact with the outside world (26). On the other hand, due to fear of impending death and the inability of the body to cooperate, patients often feel powerless and become capricious, and they often shift their anger towards their spouse because of all aspects of little things.

‘When he could not move in bed, I took care of him to feed, defaecate, and so on. He was good at losing his temper and scolding him. I was scolded and cried last time. He scolded me all day and night with an ugly tone. I have no way’. (Spouse 1)

‘Now she rarely communicates with me. She might say physical discomfort, but rarely says inner thoughts. Sometimes when she felt a little comfortable, she might occasionally say about the child, after all, the child is still relatively small, just going to the kindergarten’. (Spouse 2)

##### 3.3.2.2 Feeling emotional traction when spouses caregivers take care of patients

Patients with terminal cancer often experience inhuman physical pain and psychological torture. The spouse, as the closest daily caregiver, is often the easiest to perceive and most vulnerable to emotional backlash (28). If the patient’s pain is relieved, their spouse and family members will naturally feel at ease. If a patient is unwell or silent, they often remain awake at night with worry.

‘If he is good, of course, I also feel better. If he is sad, I cannot eat or sleep. The family is like this; if he is happy, you are happy; if he is sad, you are sadder’. (Spouse 10)

#### 3.3.3 Theme 3: Psychological feelings of spouses caregivers when they will being widowed soon

##### 3.3.3.1 Feeling deeply anxious about the patient’s inevitable death and in a high-alert state

Death anxiety is the knowledge that oneself or a loved one will die and generate a feeling of fear (21). Studies have shown that as a patient’s death approaches, the family’s anxiety increases (22). From the moment the spouse was informed that the patient was in the near-death stage, they were worried and fearful, which persisted throughout the illness. Therefore, spouses are often in a high-alert state during the near-death stage.

‘When it is another family member’s turn to take care of her, I am so tired and sleepy that I fall asleep soon after going home. However, I am often afraid that doctors will find us. Sometimes, in the hospital, I wake up as soon as the nurse comes in the sick room; I was just afraid of any bad thing’. (Spouse 2)

‘I just do not dare to sleep. I am afraid that the hospital workers or someone else will call me to say bad news’. (Spouse 3)

##### 3.3.3.2 Having obvious anticipatory grief for a patient’s approaching death

Anticipatory grief is a series of sadness reactions in which an individual expects an important person to die soon (23). When the lover is on the verge of suffering, the suffering of the spouse is no less than that of the patient (28). As death approaches their lovers, most spouses produce a series of major sad reactions because of the superposition of various emotional burdens (23). However, some spouses in the same situation experience a sense of relief and relaxation due to chronic stress and exhaustion (23).

‘Dare not think about the future, the thought of life and death; I cannot stop tears, cannot control myself. This was particularly uncomfortable. The child was admitted to university. I really dare not to think about this’. (Spouse 10)

#### 3.3.4 Theme 4: The focus of life shift, and life concept change

##### 3.3.4.1 Feeling heavy pressure and burden, and physical exhaustion

From the moment the patient was diagnosed with cancer, the spouse, closest lover, and caregiver faced various burdens and pressures. All these peaked and allowed the spouse to become exhausted during the cancer-related death period. Previous studies have shown that the most prominent pressure is the economic burden due to the long-term expenditure on cancer diagnosis and treatment (29, 30).

‘I do not make money now and have to take care of two children. We also have a big debt because he has spent more than a hundred thousand Yuan now (1 Yuan = 0.1384 dollars). It cannot always be like this when we sell all we can sell and cannot borrow any money from others’. (Spouse 9)

‘These days, he could not move, I took care of him particularly hard, there was no way. These days, I also had a fever, but I still insisted on doing so. I was very afraid that he would suddenly leave, and I would no longer see him. Even if I was very tired, I would still insist and support myself to not fall’. (Spouse 6)

##### 3.3.4.2 Reflecting on the essence of life, and changing the concept of life

In the process of caring for a patient with terminal cancer for a long time, their spouse faces the patient’s inhuman pain and the fact that they are going to die. All of these factors constantly impact their understanding of life and death. When caring for and accompanying patients, spouses are forced to slow down, reexamine themselves, relocate their gains and losses in life, and reflect on the meaning and essence of life.

‘Before she suffered cancer, I always felt that it was very important for me to work and make money. Now, I felt nothing was more important than health and that life and life were very fragile. I would not feel so deep; however, I met with my lover like this, illness developing quickly in such a short time. I suddenly felt meaningless in pursuit of myself, my work, and my career. The pursuit of yourself, your work, and your career suddenly feel meaningless’. (Spouse 2)

## 4 Discussion and conclusion

### 4.1 Discussion

Our key findings indicated that the stress response scores of the 186 spouse caregivers were 66.92 ± 21.66. There was a negative and moderate correlation between spousal stress response and spousal social support. Meanwhile, four influencing factors statistically affected spousal stress response: whether the spouse had alternative care for other dependents, spousal social support, and number of venous pathways and instruments in/on the patient’s body. Among these influencing factors, spousal social support was the best influencing factor to predict spousal stress response, followed by whether the spouse had alternative care for other dependents. Four qualitative themes were identified: (1) psychological feelings of spousal caregivers when caring for patients’ physical function, (2) psychological feelings of spousal caregivers when communicating with patients, (3) psychological feelings of spousal caregivers when they were widowed soon, and (4) the focus on life shift and life concept changes.

We found that spousal stress response scores were high (66.92 ± 21.66), which was significantly higher than those of 1,323 undergraduates as a domestic norms (53.67 ± 18.99) and 446 undergraduates (52.79 ± 19.82) (31). Meanwhile, we also found that the psychological response dimension scored the highest, followed by the physiological response, whereas the behavioural response score was the lowest. Similarly, the physical and psychological stress responses of cancer caregivers were at high levels, and the psychological stress responses are more obvious (32, 33). In terms of psychological response, spousal caregivers had a higher incidence of anxiety, depression, and other emotional problems than family caregivers or patients with cancer (34). In terms of physiological response, it was mainly due to long-term load of care work, lack of sleep and fatigue, which led to decreased immunity, insomnia, decreased appetite and other symptoms (32). Our qualitative results also further argued that, which was as following: ‘Feeling deeply distressed when spouse caregivers faced with the patient dying symptoms’, ‘Feeling heavy pressure and burden, and physical exhaustion’, and so on. Adverse stress events can cause individuals to respond negatively to psychological stress. In this study, the imminent death of a loved one was a major adverse stress event for the spouses. Compared with caregivers of patients with cancer in other periods, a series of stress reactions was more severe, and the closer the relationship with the patient, the more severe the reaction (11, 12). Especially for the spousal caregivers who had not alternative care for other dependents, low social support, more number of infusion lines and instruments in/on the patient’s body, the stress response of them was relatively strong. Therefore, clinical medical staff can take interventions to reduce the level of spousal stress response during the dying period, like improving spouse social support.

Social support is a complex structure that has long been recognised as having a direct buffering effect on the health and emotional regulation of cancer patients (35, 36). In this study, we found that spousal social support scores were above the average of 40.86 ± 8.21. The score for subjective support was the highest, and that for support utilisation was the lowest. This is because young and middle-aged patients with terminal cancer are the mainstay of both family and society, and they are also near death with serious illness and a short survival time. Relatives, family, and friends in society and work units may come to care for, visit, and help patients, and spouses feel respected, understood, and supported. However, the utilisation score of social support is the lowest, which may be related to the fact that only a few spouses can receive support. Therefore, health workers should be concerned about, support, and guide spouses of young and middle-aged patients with terminal cancer to assist them in establishing a family support service system. For example, video tools can provide knowledge about end-of-life care for patients with cancer and their caregivers (37).

Social support can indirectly buffer the effects of life events by improving individuals’ coping abilities and adaptability, thus reducing their stress response (38, 39). The stress buffer model emphasises that when an individual experiences external stress, the external support of necessary resources can effectively alleviate the possible damage caused by it (40). The results of this study supported that. We found that spouse caregivers with higher social support scores remained more likely to have poor stress response. And spousal social support was the best influencing factor to predict spousal stress response. This suggests that social support has a significant easing effect on stress responses, and family support can help caregivers balance their emotions and spirits, reduce their stress, and maintain a healthy mental state. Healthcare providers have developed intervention plans for cancer to increase spousal social support, especially from friends, and have suggested that they join support groups (41). However, little research has been conducted on the stress response and social support of spouse caregivers who care for young and middle-aged patients with terminal cancer. A previous study suggested that frequent social status assessments of cancer patients are crucial, particularly for those with poor social support (42). Meanwhile, adult patients with cancer often receive intensive care and exhibit obvious symptoms during the cancer–dying period. Incorporating palliative care into patient care may help alleviate the suffering of patients and families (43). Therefore, in addition to caring for and supporting spousal caregivers, healthcare workers should also pay attention to spouses’ social resources and encourage their relatives and friends to help their spouses in physical, mental, spiritual, and social aspects to reduce their stress response.

Care-giving behaviour can affect caregivers’ lives, rest, and sleep, and excessive and prolonged care can also affect physical health (44). This study found a significant negative correlation between family surrogate care and spousal stress response. The qualitative results emphasised ‘Feeling of heavy pressure, burden, and physical exhaustion’. Spouse 6 said that *‘These days, he could not move, I took care of him particularly hard, there was no way. These days, I also had a fever, but I still insisted on doing so. I was very afraid that he would suddenly leave, and I would no longer see him. Even if I was very tired, I would still insist and support myself to not fall’.* Spousal caregivers without family surrogate care showed the strongest responses to stress. This might be related to the fact that the patients with cancer were in the cancer-dying period, and the spouses had to take care of such patients to maintain their daily life functions and face many issues, such as whether to rescue them or give up treatment or other medical decisions, including whether to prepare for the patient’s affairs after death (11). Fu et al. found an often overlooked meandering decision-making process in which family caregivers admitted their family members with terminal cancer to inpatient hospice care (45). Similarly, Li et al. found that breast reconstruction decisions for Chinese patients with breast cancer are often complex and accompanied by conflicting decisions (46). During this period, the spouse is the most helpless and confused, and if there is no family replacement care and discussion with families, it might lead to a strong sense of helplessness, stress, and fatigue for the spouse and a decline in positive social attitudes for the patient. Therefore, healthcare workers should remind spousal caregivers to rest properly and let other family members take turns taking care of patients to reduce their workload, maintain physical and mental health, and improve their quality of care.

In this study, we found that the number of infusion pipes and instruments was the main factor affecting spousal stress response. The reasons were as follows: First, the more pipelines on patients in the cancer-dying period, the more difficult it is for family members and nurses to take basic care of patients, such as turning over, changing sheets and clothes, and disposing excreta, thus aggravating the care burden. Our qualitative results further support ‘Feeling heavy pressure, burden, and physical exhaustion’. Spouse 9 said that *‘I do not make money now and have to take care of two children. We also have a big debt because he has spent more than a hundred thousand Yuan now (1 Yuan = 0.1384 dollars). It cannot always be like this when we sell all we can sell and cannot borrow any money from others’.* Second, young and middle-aged patients with cancer were originally a sound family core figure; however, now the body is filled with pipes and machines, which can easily cause the spouse to recall the patient’s past healthy state, sigh, and emote, thus aggravating the production of a stress response. The qualitative results also indicated ‘Feeling deeply fearful when spouse caregivers touched the machine and pipeline on the body of patients with terminal cancer’. Spouse 1 said that *‘My husband now has an abdominal tube on the left side of his abdomen, a thoracic tube in the right of the chest, and a PICC tube on the hand for venous transfusion. Sometimes, a lien needle tube is placed on the hand during blood transfusion. It is scary that he is filled with tubes in the body. When we wipe him, we are afraid of touching it off or distorting it off’.* Therefore, when nursing patients are near death, healthcare workers should strictly follow the standard care of drainage and infusion pipes, sort out the instruments in use, and reduce the stimulation and influence of these pipes on spousal caregivers.

Our study aimed to explore the death care experiences of spousal caregivers of young and middle-aged patients with terminal cancer, to further investigate their stress responses. Four qualitative themes are identified. Similarly, a previous qualitative study on the decision-making process of Chinese family caregivers of patients with terminal cancer showed that they underwent a winding and socioculturally-mediated four-stage process (45). This previous study indicated that caregivers’ attitudes towards death and their family members with terminal cancer expressed care needs that influenced the aftermath. It also suggests that training healthcare workers in cultural competence, developing an effective hospice referral system, and delivering socioculturally acceptable death education are critical interventions for facilitating better decision-making experiences (45).

### 4.2 Strength and limitations

The strengths of this study include a prospective and mixed-methods design to investigate the stress responses of spouse caregivers, explore their death care experience, and focus on spouse caregivers of young and middle-aged patients with terminal cancer. Our study has some limitations. First, owing to the influence of time and manpower, we only selected patients from one hospital in China, which might have had sample limitations and bias. Second, the sample size of this study was small. Third, social support was highlighted as an important factor in this study; however, social support had only been measured via quantitative method and had not been explored in depth via qualitative method. That might result in a superficial understanding of the impact of spousal social support on spousal stress response. Finally, the length of the dying period of cancer patients was different; therefore, the time window of data collection was not sufficiently concentrated and prone to bias.

### 4.3 Conclusion

Overall, spousal stress response was obvious and high, and it statistically affected alternative caregivers for spouses, spousal social support, and the number of venous pathways and instruments in/on the patient’s body. Medical personnel should pay attention to the caregivers’ physical and mental conditions and needs when caring for patients. It aims to provide human care, social resources, social support, and other aspects of information; help reduce the degree of stress response; and improve physical and mental health and care abilities. However, some caregivers did not receive sufficient support and understanding, suggesting that more attention should be paid to the psychological status and quality of life of this group in future research. Simultaneously, we need to explore ways to improve social support levels through effective intervention measures to alleviate caregivers’ stress reactions and improve the quality of life of patients and their families.

### 4.4 Practice implications

Clinical medical staff must constantly improve their professional knowledge and skills, relieve the pain of patients with terminal cancer as much as possible, and maintain their dignity. In addition, clinical medical staff should assess the internal support resources of their spouses and the living habits of the patients during communication between their spouses and family members. According to the patients’ preferences, clinical medical staff can assist them in asking for help from social workers, volunteers, and religious personnel to expand the social support system. It is more effective to take preventative measures before death rather than offering counselling after death. Clinical medical staff can also provide death education to their spouses and family members at appropriate times, according to the actual situation of the patients, that can more help them to deal with patients’ death in an orderly manner. Moreover, with the development of the Internet era and information technology, the Wechat (微信) public account platform or Wechat mini program also can be used to set up death procedures and funeral service procedures to help their spouses and other families prepare for the future.

## Data Availability

The raw data supporting the conclusions of this article will be made available by the authors, without undue reservation.
